# Ludwig’s Angina

**DOI:** 10.7759/cureus.1588

**Published:** 2017-08-21

**Authors:** Stella Pak, David Cha, Chloe Meyer, Christine Dee, Adam Fershko

**Affiliations:** 1 Internal Medicine, Kettering Medical Center; 2 Wright State University Boonshoft School of Medicine

**Keywords:** ludwig’s angina, odontogenic infection, antibiotics, steroids

## Abstract

Ludwig’s angina is a diffuse cellulitis in the submandibular, sublingual, and submental spaces, characterized by its propensity to spread rapidly to the surrounding tissues. Early recognition and treatment for Ludwig’s angina are of paramount importance due to the myriad of complications that can occur in association with Ludwig’s angina. Known complications of Ludwig’s angina include carotid arterial rupture or sheath abscess, thrombophlebitis of the internal jugular vein, mediastinitis, empyema, pericardial effusion, osteomyelitis of the mandible, subphrenic abscess, aspiration pneumonia, and pleural effusion. By reporting a case of Ludwig’s angina, we hope to raise the awareness in our medical community for this rare clinical entity.

This case describes a 54-year-old woman with Ludwig’s angina that evolved from a chronic odontogenic infection. She presented with perioral swelling with the involvement of bilateral submandibular and sublingual areas, accompanied by excruciating pain, chills, fever, and vomiting. She was treated with clindamycin and cefoxitin for infection and vigorously hydrated.

This case is exemplary for the successful management of this potentially lethal clinical condition. Our early recognition and aggressive treatment helped to prevent complications from Ludwig’s angina.

## Introduction

Ludwig’s angina is a diffuse cellulitis of the submandibular, sublingual, and submental space, characterized by its propensity to spread rapidly to the surrounding tissues [1]. Early recognition and treatment for Ludwig’s angina are of paramount importance due to the myriad of complications that can occur in association with Ludwig’s angina. Possible complications of Ludwig’s angina are airway obstruction, carotid arterial rupture or sheath abscess, thrombophlebitis of the internal jugular vein, mediastinits, empyema, necrotizing fasciitis, pericardial effusion, osteomyelitis, subphrenic abscess, aspiration pneumonia, and pleural effusion [2-4]. Ludwig’s angina is a potentially lethal infection with a mortality of 8% [1]. Ludwig’s angina usually evolves from odontogenic infections, a penetrating injury in the floor of the mouth, osteomyelitis or fracture of the jaw, otitis media, tongue piercing, sialdenitis, or silaolithiasis of the submandibular glands [3]. Herein, we report a case of a middle-aged woman with Ludwig’s angina that evolved from a chronic odontogenic infection.

## Case presentation

A 54-year-old woman presented with multiple untreated dental caries and a molar tooth fracture. She reported that the molar tooth fracture on the right side had not been treated for a year due to the lack of dental insurance. A week prior to her initial presentation, she suddenly developed acute atraumatic pain in the right side of her jaw. The pain rapidly escalated as days passed. On the fifth day, she ingested approximately 20 tablets (200 mg per tablet) of ibuprofen for pain relief, but her pain persisted with increasing severity. On the seventh day, she became unable to open her mouth due to excruciating pain radiating to both sides of her chin. She was unable to tolerate oral intake because of the pain associated with opening her mouth. She also developed a severe headache in the frontal and maxillary regions. Moreover, she reported chills, nausea, vomiting, and dysphagia. However, she denied chest pain, dyspnea, diarrhea, or pain in the upper face or shoulders.

**Figure 1:**
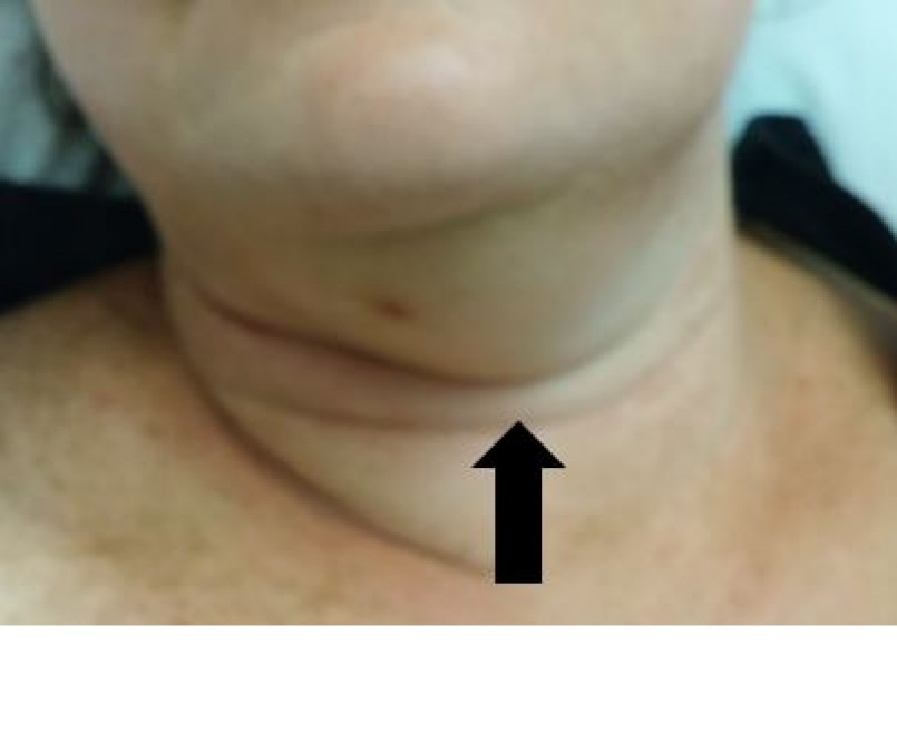
Prominent swelling (arrow) in the submandibular and sublingual areas in Ludwig’s angina

Her blood pressure was 93/47 mmHg with a heart rate of 96/min, a respiration rate of 18/min, body temperature of 103 °F, and an oxygen saturation percentage of 96% on room air. She was diaphoretic and weak. Submandibular adenopathy and extraoral swelling with bilateral involvement of submandibular and sublingual region were prominent on physical examination (Figure 1). The swelling was indurated, non-fluctuant, and exquisitely tender. She had leukocytosis of 13,700/uL. A computerized tomography (CT) scan of her neck visualized cervical lymphadenopathy on both sides but did not show any evidence of abscess or bone erosion.

Our patient was vigorously hydrated with intravenous (IV) fluid and started on clindamycin. On the following day, her antibiotic regimen was changed to cefoxitin for improved penetrance to the infection site in the oral cavity. She was also symptomatically treated with morphine, ketorolac, and ondansetron. On the second day, she was given IV methylprednisolone.

On the third day of her hospitalization, she reported improvement in pain and dysphagia. Her vital signs were all within the normal range and her white blood cells (WBC) count trended down to 8.2. She was given piperacillin-tazobactam and subsequently discharged on amoxicillin-clavulanate for seven days.

## Discussion

Ludwig angina, first described by Wilhelm Fredrick von Ludwig in 1836, is an infection of the sublingual space and submylohyoid space. It is bilateral and can spread rapidly, secondary to being compartmentalized within the submandibular space [5]. A simple odontogenic problem can quickly turn fatal because of numerous critical complications, such as airway edema. As represented in this case, patients can present with exquisite pain that could yield profound sepsis if not promptly treated with antibiotics and other critical care measures, such as maintaining the airway and aggressively hydrating [6].

Caregivers should be wary of patients with a history of dental infections, especially of the second or third mandibular molars. The molar fracture that plagued the woman in this case was ultimately the initiating event that led to infection. Alternatively, spread from peritonsillar abscesses or suppurative parotitis has been documented. Ludwig’s angina is commonly polymicrobial, keeping in mind the normal flora of the oral cavity. Commonly involved organisms are Streptococcus viridans and anaerobes like Fusobacterium nucleatum, Peptostreptococcus species, and Actinomyces species [7]. On physical exam, this patient had adenopathy, although that is not always the case. Other findings may be “woody” induration with crepitus and an erythematous floor of the mouth. By combining the physical exam findings, a clinical history, CT imaging, and possibly a Gram stain of aspirated fluid from the site, a timely diagnosis can be made so that appropriate treatment can begin before the serious complications previously discussed take place [8].

Airway management is the first step in the medical management of Ludwig’s angina as airway compromise is the leading cause of death. Previous findings in a retrospective review advocated the use of elective awake tracheostomy as a much safer method than endotracheal intubation [9]. Early antibiotic therapy is of critical importance for successful treatment. Penicillin G, metronidazole, or clindamycin are good choices as initial coverage [9]. Moreover, intravenous steroids and nebulized adrenaline use have been shown to allow for easier intubation avoiding tracheostomy or cricothyroidotomy and allowing for increased penetration of antibiotics into the fascial spaces by reducing edema and cellulitis [10]. However, emergency cricothyroidotomy or tracheostomy is indicated in patients seen in the late stages of this disease. Surgery is indicated for patients who develop abscesses and are unresponsive to antibiotics and medical management. This is usually accomplished by decompressing the submental, submandibular, and sublingual spaces by external incision and drainage [9].

## Conclusions

Early recognition and treatment of Ludwig’s angina are critical as life-threatening complications, such as airway obstruction and necrotizing fasciitis, can occur from this clinicopathological entity. With increasing availability of antibiotics and improvements in oral hygiene, Ludwig’s angina has become a rare emergency in the United States. The rarity of Ludwig’s angina and fast-paced work flow in modern health care facilities makes it difficult to quickly recognize this rare condition. The aim of this case report is to improve the awareness of Ludwig’s angina in our health care community.
